# Microfocusing at the PG1 beamline at FLASH

**DOI:** 10.1107/S1600577515023127

**Published:** 2016-01-01

**Authors:** Siarhei Dziarzhytski, Natalia Gerasimova, Rene Goderich, Tobias Mey, Ruben Reininger, Michael Rübhausen, Frank Siewert, Holger Weigelt, Günter Brenner

**Affiliations:** a DESY, Notkestrasse 85, 22067 Hamburg, Germany; b European XFEL GmbH, Albert-Einstein-Ring 19, 22761 Hamburg, Germany; c University of South Florida, USA; d Laser Laboratorium Göttingen eV, Hans-Adolf-Krebs-Weg 1, 37077 Göttingen, Germany; e Advanced Photon Source, Argonne National Laboratory, Argonne, IL 60439, USA; f University of Hamburg and Center for Free-Electron Laser Science, Notkestrasse 85, 22607 Hamburg, Germany; g Institute for Nanometre Optics and Technology at Helmholtz Zentrum Berlin/BESSY II, Albert-Einstein-Strasse 15, 12489 Berlin, Germany

**Keywords:** free-electron laser, Kirkpatrick–Baez mirror system, microfocus, plane-grating monochromator beamline

## Abstract

The Kirkpatrick–Baez (KB) refocusing mirror system installed at the PG1 branch of the plane-grating monochromator beamline at the soft X-ray/XUV free-electron laser in Hamburg (FLASH) is designed to provide tight aberration-free focusing down to 4 µm × 6 µm full width at half-maximum (FWHM) on the sample. Such a focal spot size is mandatory to achieve ultimate resolution and to guarantee best performance of the vacuum-ultraviolet (VUV) off-axis parabolic double-monochromator Raman spectrometer permanently installed at the PG1 beamline as an experimental end-station. The vertical beam size on the sample of the Raman spectrometer, which operates without entrance slit, defines and limits the energy resolution of the instrument which has an unprecedented design value of 2 meV for photon energies below 70 eV and about 15 meV for higher energies up to 200 eV. In order to reach the designed focal spot size of 4 µm FWHM (vertically) and to hold the highest spectrometer resolution, special fully motorized in-vacuum manipulators for the KB mirror holders have been developed and the optics have been aligned employing wavefront-sensing techniques as well as ablative imprints analysis. Aberrations like astigmatism were minimized. In this article the design and layout of the KB mirror manipulators, the alignment procedure as well as microfocus optimization results are presented.

## Abstract 2

The Kirkpatrick–Baez (KB) refocusing mirrors unit at the PG1 beamline at FLASH has been newly designed, developed and fully commissioned. The vertical focal size of the KB optics is measured to be 5.8 ± 1 µm FWHM and the horizontal 6 ± 2 µm FWHM; astigmatism has been minimized to below 1 mm between waist positions. Such a tight focus is essential for the VUV double Raman spectrometer as it serves as an entrance slit for the first monochromator and defines its resolution to a very large extent. The Raman spectrometer is a permanent end-station at the PG1 beamline, dedicated to inelastic soft X-ray scattering experiments.

## 1. Introduction   

FLASH is a single-pass free-electron laser (FEL) whose working principle is based on the self-amplified spontaneous emission (SASE) process (Ackermann *et al.*, 2007 ▸). Operating as a user facility since 2005, FLASH provides XUV and THz radiation to user experiments at five beamlines (PG1, PG2, BL1, BL2, BL3) (Tiedtke *et al.*, 2009 ▸). The FEL beam is switched between beamlines by plane grazing-incidence mirrors. The so-called PG beamline is equipped with a high-resolution plane-grating monochromator (PGM) of SX700 type (Petersen, 1982 ▸; Riemer & Torge, 1983 ▸) and employs two beamline branches, namely PG1 and PG2 (Martins *et al.*, 2006 ▸; Gerasimova *et al.*, 2011 ▸), used alternatively. While PG2 serves as an open port for various types of user experiments, PG1 has a VUV double-stage Raman spectrometer installed as a permanent end-station, dedicated to inelastic soft X-ray scattering (IXS) experiments. This unique instrument covers a photon energy range from 20 to 200 eV with unprecedented energy resolution of about 2–15 meV (design values) for the entire spectral range and allows a strong suppression of the elastic line (Rusydi *et al.*, 2014 ▸). Since both off-axis parabolic Raman monochromators disperse the scattered radiation in the vertical direction and the first monochromator stage does not have an entrance slit, the maximum achievable resolution of the instrument is in principle limited by the vertical spot size of the FEL beam on the sample. Hence, the optimization and alignment of the PG1 beamline focus is indispensable to meet the ultimate resolution of the Raman spectrometer.

Many third-generation light sources like storage-ring-based synchrotrons use refocusing optic systems in a configuration developed by Kirkpatrick and Baez in 1948 (Kirkpatrick & Baez, 1948 ▸) with or without modifications like mirror bending or staging (Yashchuk *et al.*, 2013 ▸). A Kirkpatrick–Baez pair (KB) of plane elliptical mirrors separates focusing in sagittal and tangential planes and thus provides wavelength-independent spherical and astigmatic aberration-free focusing of radiation. Nowadays, also fourth-generation light sources like FELs incorporate KB-focusing configurations in their beamline design (Raimondi *et al.*, 2014 ▸; Siewert *et al.*, 2012 ▸; Tono *et al.*, 2013 ▸). As will be shown in the following, the KB system installed at the PG1 beamline provides sub-10 µm microfocusing, thus making high-resolution Raman scattering experiments possible.

## 2. The PG1 beamline   

**Figure 1:**
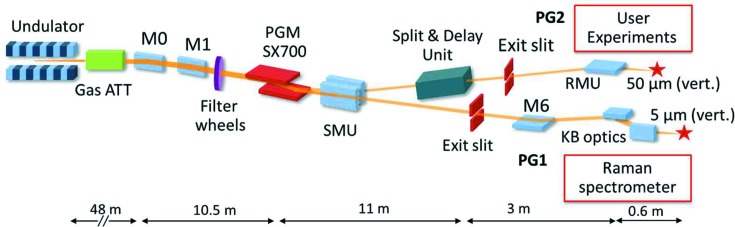
Overview of the plane-grating monochromator (PGM) beamline at FLASH. Behind the monochromator the beam is guided either to the PG1 or PG2 beamline branch by a cylindrical switching mirror unit (SMU). At PG1 a plane mirror M6 lifts the dispersed beam up to the Kirkpatrick–Baez mirror unit which refocuses the beam onto the sample of the permanently installed Raman spectrometer endstation. Only the focus sizes in the vertical direction for both beamlines are indicated in the figure.

The PG1 monochromator beamline branch is schematically shown in Fig. 1 ▸. Briefly, the PG1 beamline consists of eight optical elements. A plane mirror M0 switches the FEL beam between different beamlines of FLASH, followed by a toroidal mirror M1 which focuses the beam in the horizontal direction to a focal spot that lies 1 m before the PG monochromator exit slit and collimates the FEL beam in the vertical direction. Passing the PG monochromator, the vertically dispersed FEL beam is then focused by the cylindrical switching mirror unit (SMU) vertically onto the exit slit position. A plane mirror M6 lifts the beam up which was needed for installation of the Raman spectrometer. The KB pair installed at the end of the PG1 beamline focuses the beam onto the sample in the experimental chamber of the Raman spectrometer. The PG1 monochromator exit slit (SMU vertical focus) is refocused by the vertically focusing mirror of the KB pair (M7), while the horizontal focus from the toroidal mirror M1 is refocused by the horizontally focusing mirror of the KB pair (M8).

## 3. Design and development of the KB refocusing unit   

The optical design of the KB refocusing mirror unit was carried out by the company Scientific Answers and Solutions. Mirrors and mechanics were manufactured in the collaboration of DESY with University of Hamburg (AG Rübhausen) and several companies (BesTec, Jenoptik, Carl Zeiss). The mirrors have been characterized using the Nanometer Optical Component Measuring Machine (Siewert *et al.*, 2004 ▸) at the BESSY-II Optics Laboratory of the Helmholtz Zentrum Berlin (Siewert *et al.*, 2010 ▸). In order to provide precise *in situ* mirror alignment options the KB mirror system is equipped with in-vacuum manipulators. The necessary resolution and tolerances of the manipulators were guided by simulations carried out within the *XOP SHADOW* package (Cerrina & Sanchez del Rio, 2010 ▸) (see below). As mentioned before, the KB refocusing optics consist of two mirrors: M7 which deflects the beam in the vertical direction and re-images the vertical PGM slit onto the sample position, and mirror M8 which refocuses in the horizontal direction. The incident angle of both focusing elements measured from their surface is 6°. Fully in-vacuum motorized mirror holders provide the needed angle resolution up to 0.003° and movement resolution of 30 µm for the most crucial degrees of freedom (DoF) like pitch and roll angles and translations along the beam as well as along the mirror normal, respectively.

**Figure 2:**
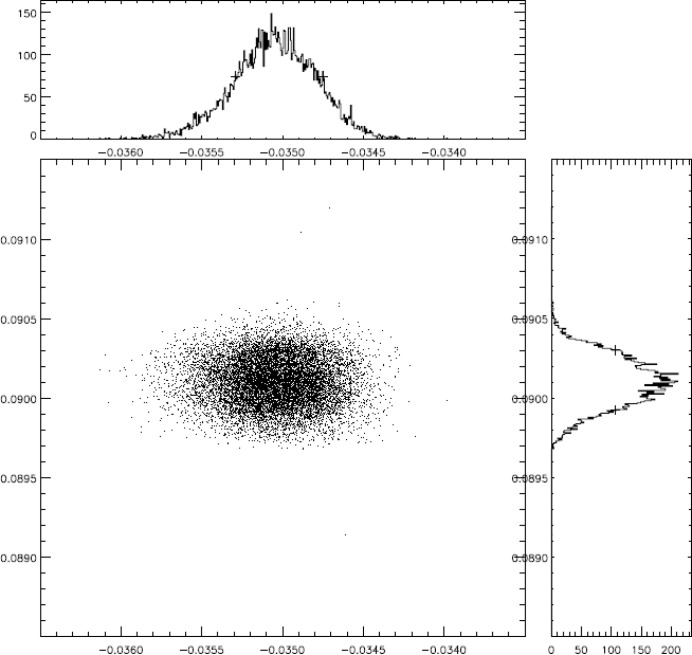
Focal spot size (FWHM) of the KB optics simulated in *SHADOW*. The focus size is 4.1 µm × 6.7 µm (vertical × horizontal). The simulation was carried out for a 13.5 nm FEL photon wavelength, the PG monochromator in first order (*c*_ff_ = 2) and an exit slit width of 20 µm. Surface roughness and slope error of each focusing optical element of the beamline were taken into account.

The whole PG1 beamline including the KB pair has been ray-traced *via* *XOP SHADOW* simulation software. Parameters of the PG1 focusing optical elements are given in Table 1 ▸. The source was modelled with a diameter size of ∼160 µm and a divergence of ∼150 µrad with Gaussian spatial and angular distribution. An example of the simulated focal spot size is shown in Fig. 2 ▸. The theoretical focal spot size is calculated to be of 4.1 µm FWHM vertically and 6.7 µm FWHM horizontally. The simulations were performed at a FEL wavelength of 13.5 nm, the PG monochromator in the first order, fixed-focus-constant *c*_ff_ = 2 and exit slit width of 20 µm. Furthermore, the surface roughness and slope error of each focusing optical element of the beamline was taken into account.

**Figure 3:**
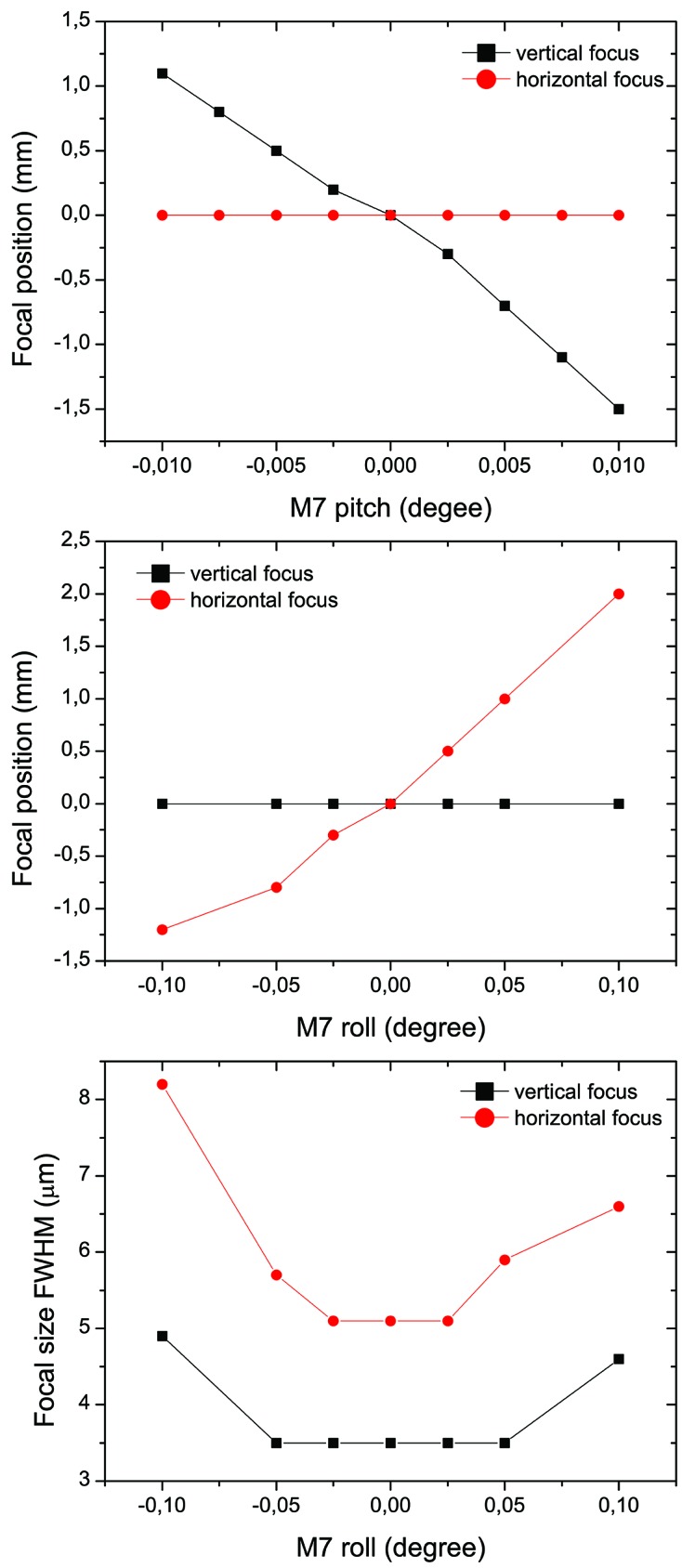
Simulated (*SHADOW*) effect of M7 mirror pitch and roll angle misalignment on its focusing properties. The focus position variation is estimated relative to its nominal position of 400 mm downstream from the pole of the M8 mirror when the M7/M8 mirrors are aligned. For further explanation see the text.

Within these simulations the influence of each DoF (pitch, roll, yaw angles as well as translation along the mirror normal and along the beam) of the KB pair mirrors on their focusing properties was estimated to provide manipulator tolerances and required motor resolution. Our studies found the strongest effect on the focusing performance and aberrations from the mirror misalignment in pitch and roll angles. A summary is given in Table 2 ▸. The effect of the pitch and roll angle mis­alignment is shown in Fig. 3 ▸. Here, the focal position was calculated as a function of the rotation of the vertically focusing M7 mirror around the aforementioned angles. In the graph, the ’zero focal distance’ is the nominal KB focal distance 400 mm downstream measured from the pole of the M8 mirror. The derived required resolutions to ensure optimum alignment options for all DoF are given in Table 2 ▸.

**Figure 4:**
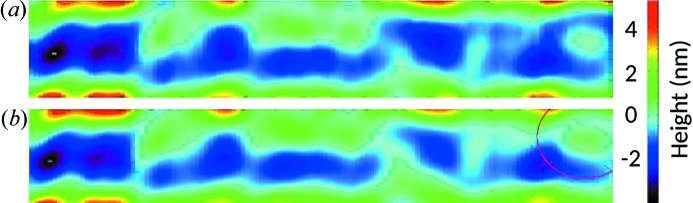
Measured M7 mirror surface map of aperture size 95 mm × 8 mm in two states: (*a*) free state, (*b*) clamped state. The red circled area indicates the area affected most by clamping. The height is shown on the right by the color bar.

**Figure 5:**
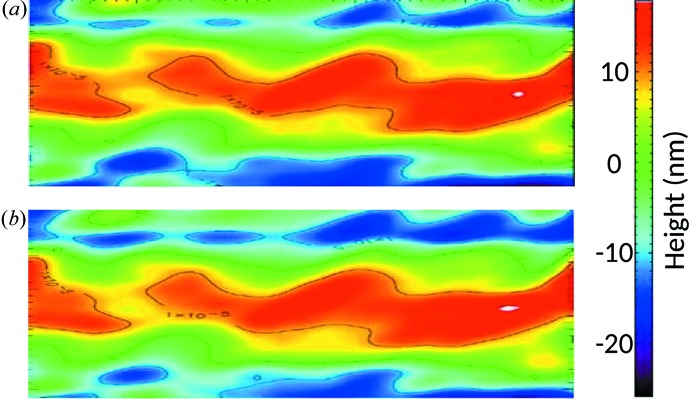
Measured M8 mirror surface map of aperture size 50 mm × 25 mm in two states: (*a*) free state, (*b*) clamped state. The height is shown on the right by a color bar.

It is well known that ultra-precise reflective optical elements have to be mounted with special care to avoid mis-shaping and to provide optimal performance at the beamline (Siewert *et al.*, 2011 ▸). Already small surface distortions of several tens of nanometres and certain space frequencies (36 mm^−1^ and 16 mm^−1^ for M7 and M8 mirrors, respectively) can increase the spot size by 20%. The effect of the mechanical clamping of the manipulators on the KB mirror surface was therefore investigated at the Helmholz Zentrum Berlin BESSY-II/INT/Optical Metrology Laboratory by two-dimensional slope mapping employing the BESSY-NOM (Siewert *et al.*, 2014 ▸). The mirror topography in terms of height was gained by integration of the slope data. The mirrors were measured in the free (unclamped) as well as in the mounted state. Mapping of, for example, the M7 mirror surface was performed in the sagittal direction (d*x* = 0.5 mm in the sagittal and d*y* = 1 mm in the longitudinal directions). Fig. 4 ▸ shows the M7 central aperture section of 95 mm × 8 mm. Only small figure deformations in the range of 2 nm peak-to-valley and less were found. Such a small effect is stipulated by the mirror thickness of 45 mm and clamping mechanism, a U-shape frame positioned 30 mm below the optical surface of the mirrors in a special cavity and fixed to the mirror *via* three ball-head screws. The results of the M8 mirror surface measurements as a comparison of two different cases in analogy to the M7 mirror are given in Fig. 5 ▸. No visible changes were obtained in the *SHADOW* simulations with the mirrors in the unclamped or clamped cases.

**Figure 6:**
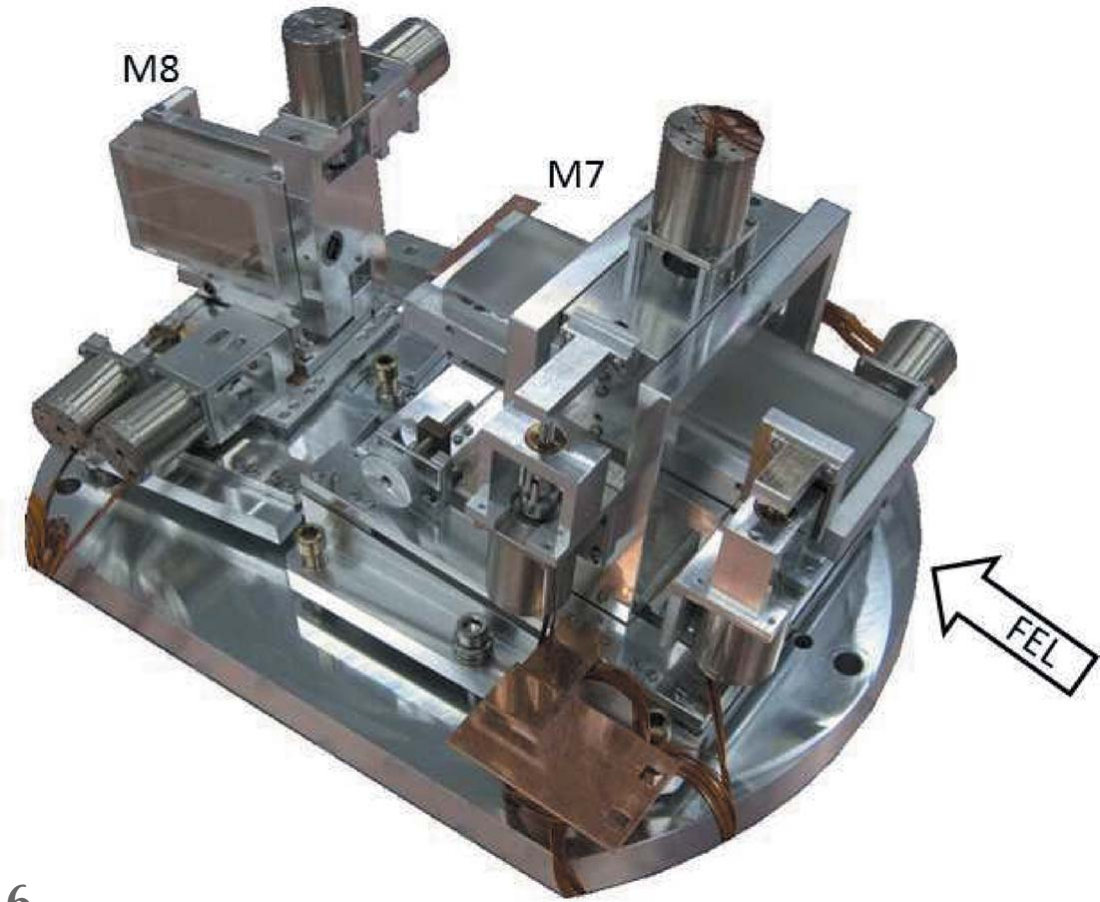
Overview of the KB mirror unit. The in-vacuum mirror manipulators are fully motorized and allow *in situ* alignment of the KB pair.

The final KB pair mount is shown in Fig. 6 ▸. The measurements of the mirror movement accuracy and reproducibility were performed by means of an optical autocollimator (ELCOMAT3000, MÖLLER-WEDEL OPTICAL GmbH) and, complementary to it, by measuring the displacement of a reflected focused He–Ne laser beam on the mirror at 2 m distance on a CCD camera (Basler scA1300-32fm, pixel size 3.75 µm) with 0.001° accuracy. The fiducialized KB unit was installed at the beamline and pre-aligned to its nominal position with the help of the survey group MEA-2 DESY using a laser tracker (Leica LTD800) and an alignment laser implemented in the PG beamline.

## 4. *In situ* KB pair focus optimization using FEL radiation   

The KB focus optimization and characterization was performed using two techniques: (i) wavefront determination as well as aberration determination employing a compact Hartmann sensor from which beam parameters like beam width, divergence, waist diameter, Rayleigh length and waist position (focus) can be reconstructed (Schäfer & Mann, 2002 ▸); and (ii) ablative imprints studies (Liu, 1982 ▸; Chalupský *et al.*, 2010 ▸). The Hartmann sensor was jointly developed by DESY and the Laser-Laboratorium Göttingen (LLG) and was available on site as a ready-to-use online diagnostics (Flöter *et al.*, 2010 ▸). Wavefront sensing (WFS) for beam characterization and beamline alignment has already been successfully used at different FEL facilities like, for example, FLASH and FERMI (Keitel *et al.*, 2016 ▸; Raimondi *et al.*, 2014 ▸). For the ablative technique we had developed a special diagnostics tool which allows determination of *in situ* focus imprint sizes along the caustic curve with 2.5 µm resolution. In addition, the instrument provides the possibility to estimate focus sizes by means of fluorescence imaging using a Ce:YAG crystal and a CCD camera with 12 µm resolution. A full review of the instrument is given by Gerasimova *et al.* (2013 ▸).

The KB focus characterization was performed for the PG monochromator set to both zero and first-order diffraction modes at FEL wavelengths of 6.5 nm, 13.5 nm and 25.8 nm, always working in single-pulse mode. Two complementary methods have been used for mainly two reasons. Firstly, wavefront measurements are carried out behind the beam waist (indirect method) and the reconstructed beam profile parameters, like, for example, the focus size, in principle depends on the degree of spatial coherence (Singer *et al.*, 2012 ▸; Flöter *et al.*, 2010 ▸). Since the coherence of the beam cannot be directly monitored and thus the influences of partial coherence are not taken into account in the evaluation, the results for focus size might suffer from an underestimation. On the other hand, the ablative imprints approach tends to overestimate the beam size for non-Gaussian beams, in particular when the intensity of the photon pulse is too high, such that the ablation process is not scaled linearly with the intensity and the beam profile has a complicated shape. Therefore both diagnostic techniques are used for focus characterization in a complementary fashion. Secondly, for IXS studies with the VUV–Raman spectrometer the PG monochromator is typically operated in dispersive high-resolution mode (first order or higher diffraction order). The photon flux which is one order (or more) of magnitude lower in the first order compared to zero order prevents single-shot ablative measurements. Therefore, wavefront sensing needs to be used to optimize and characterize quantitatively the PG1 focus in terms of its size, position and aberrations present. To complementary confirm the results from the imprint studies, wavefront measurements have also been carried out for zero-order monochromator operation. As will be shown in the following sections, the two techniques provide quantitative results which are in a very good agreement with each other.

### 4.1. Focus characterization by ablative imprints and WFS measurements (monochromator in zero order)   

#### 4.1.1. Ablative imprints measurements   

**Figure 7:**
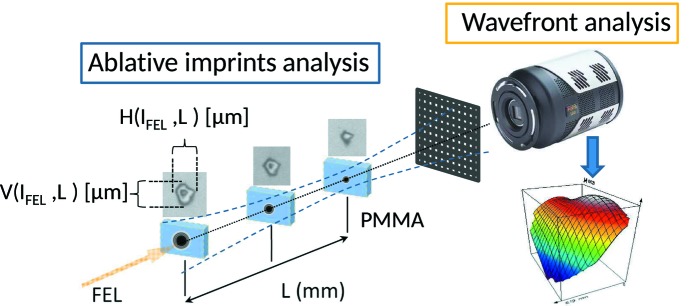
Experimental setup for KB focus characterization. Ablative imprint measurements and wavefront analysis were employed in a complementary fashion. The distance from the focus to the Hartmann plate was about 1000 mm; that from the Hartmann plate to the ICCD is 250 mm.

**Figure 8:**
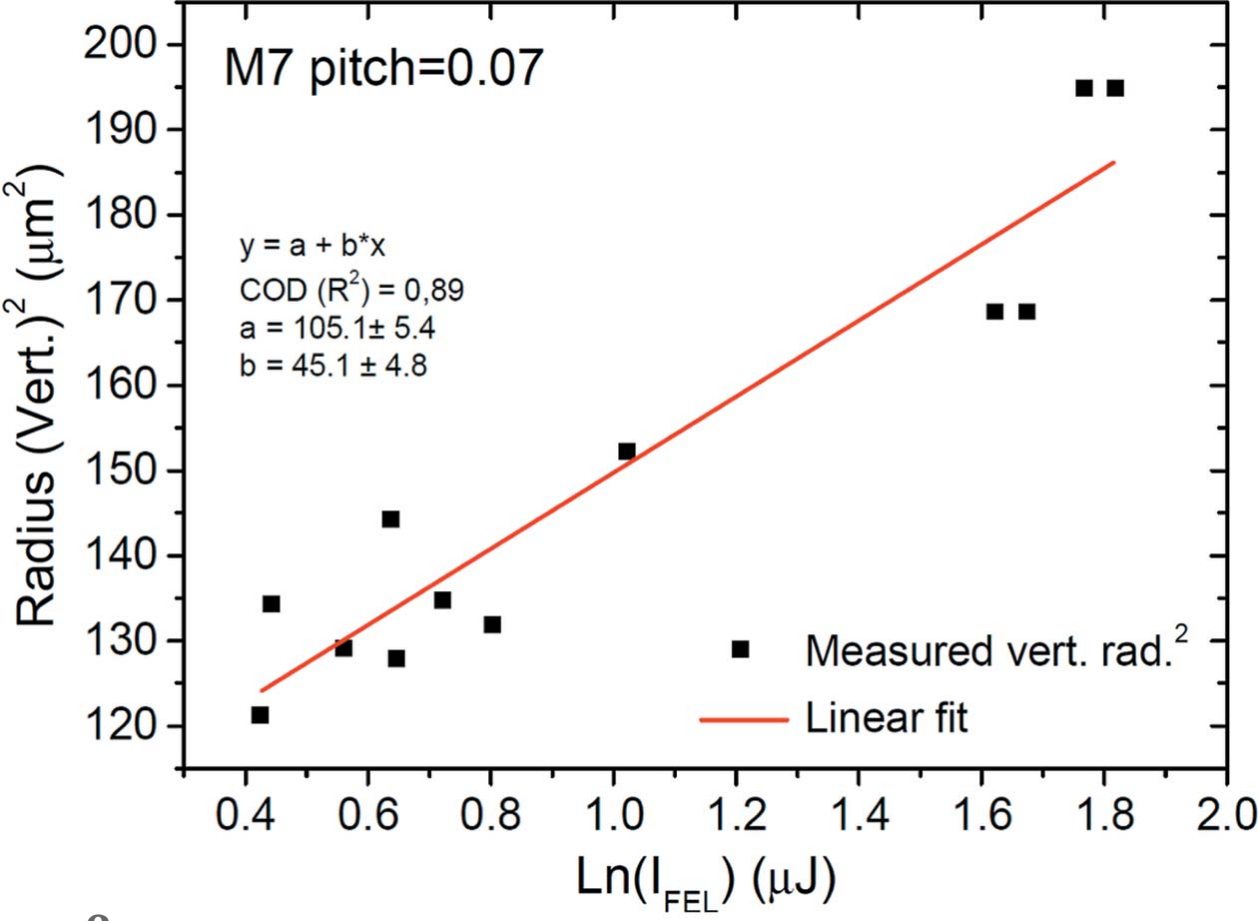
Deduction of the vertical FEL focal size from measured PMMA imprint sizes (vertical radius). The linear fit of the semilog plot of measured radius^2^ as a function of FEL pulse intensity Ln(*I*) leads to a slope which is proportional to the focus size. Here the longitudinal position includes an offset of +10 mm relative to the nominal one. The slope in this figure corresponds to a vertical focal size of (11.1 ± 0.8) µm FWHM.

Ray tracing studies on the sensitivity/influence of each mirror DoF on the KB pair focusing properties reduced the relevant parameter space for KB mirror alignment to only three parameters, namely pitch, roll and translation in the direction of the mirror normal. For the KB system alignment, single-shot ablative imprints were taken at five longitudinal positions along the focused FEL beam (*z*-scan) employing the aforementioned diagnostics chamber installed at the nominal PG1 focus position. The PG1 monochromator exit slit was kept open. PMMA [poly(methyl methacrylate) coated Si wafers (Silson UK; 20 mm × 10 mm Si wafers, 1 µm, 2.5 µm and 5 µm PMMA coated] were used as samples for the imprints. The experimental setup is schematically shown in Fig. 7 ▸. For each *z*-position, imprints at five FEL intensities with maximum pulse energies of ∼100 µJ, varied by solid state filters over a range of 10% to 0.5% transmissions, were taken. Furthermore, in order to take into account shot-to-shot fluctuations of the SASE FEL beam, five shots per given intensity and mirror setting were recorded. In total about 3000 imprints were analyzed *in situ* with the diagnostics chamber long-range microscope during the focus optimization process. Fig. 8 ▸ shows the deduction of the FEL focus size after inspection of the PMMA imprint sizes in the *X* and *Y* directions resulting from single-shot irradiation at different FEL intensities. The linear fit of this semilog plot of the beam radius as a function of FEL pulse intensity leads to a slope which is proportional to the focus size. Details of the analysis method are given by Liu (1982 ▸) and Chalupský *et al.* (2010 ▸).

**Figure 9:**
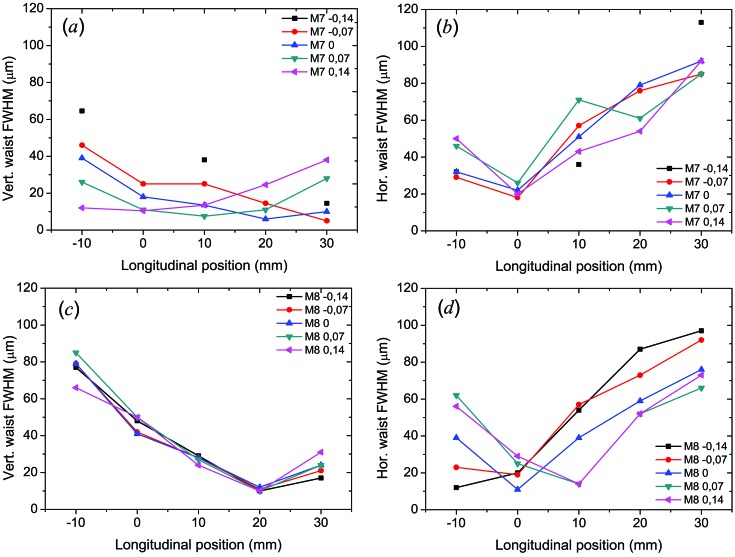
Focusing properties of the KB optics as a function of M7 pitch variation (*a*, *b*) and M8 pitch variation (*c*, *d*). The graphs show the longitudinal position of the vertical and horizontal foci together with the corresponding waist size in FWHM. For each mirror pitch variation the corresponding other mirror setting was kept fixed. Measurements were performed at 13.5 nm with the PG monochromator in zero order.

**Figure 10:**
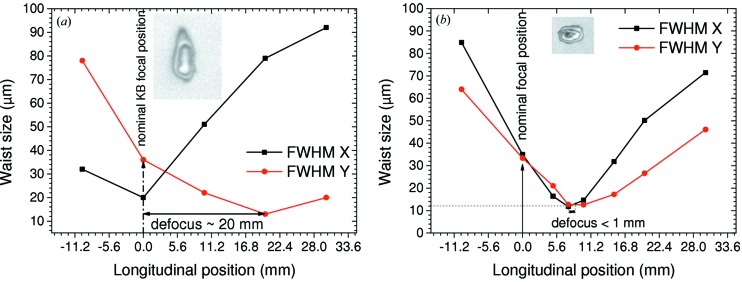
Longitudinal positions and sizes of the KB vertical and horizontal foci before (*a*) and after (*b*) the alignment in zero-order monochromator operation. The insets show the shape of a single-shot imprint as an example. Measurements were carried out at 13.5 nm. The maximum FEL pulse energy was ∼100 µJ but has been attenuated to 10–0.5% transmission during the measurement series. Further details are given in the text.

The results of M7 and M8 mirror pitch variation are given in Figs. 9 ▸ and 10 ▸, respectively. Figs. 9(*a*) and 9(*b*) ▸ clearly show that the longitudinal position of the vertical focus varies as a function of M7 pitch angle while the horizontal focus position does not change. Correspondingly, the same is valid for variation of the M8 pitch, keeping M7 fixed [Figs. 9(*c*) and 9(*d*) ▸]. The fact that under the M7 pitch variation (which should alter the vertical focus position only) the position of the horizontal focus does not change is a clear sign that horizontal and vertical foci are perfectly decoupled and thus can be overlapped to reduce an intrinsic astigmatism of the PG1 beamline introduced by the toroidal M1 mirror prior to the monochromator (see Fig. 10 ▸). There, the measured horizontal and vertical focus spot sizes along the caustic curve are plotted. The diameter of the focal spot was determined to be (11.1 ± 0.8) µm FWHM in the vertical and (12 ± 1.5) µm FWHM in the horizontal direction. Obviously, these sizes are larger than the theoretically achievable design value of approximately (4 × 6) µm. This is because by operating the monochromator in zero order the KB focus size is stipulated by the FEL source size, position and coherence. Simulations in *SHADOW* cannot take into account the latter effect. However, for Raman scattering experiments with the PG monochromator working in first or higher diffraction order the source size and position for the KB vertically focusing mirror will be fixed by the monochromator exit slit. Under such conditions a smaller vertical spot size is expected (see below).

#### 4.1.2. Focus characterization by wavefront measurements   

**Figure 11:**
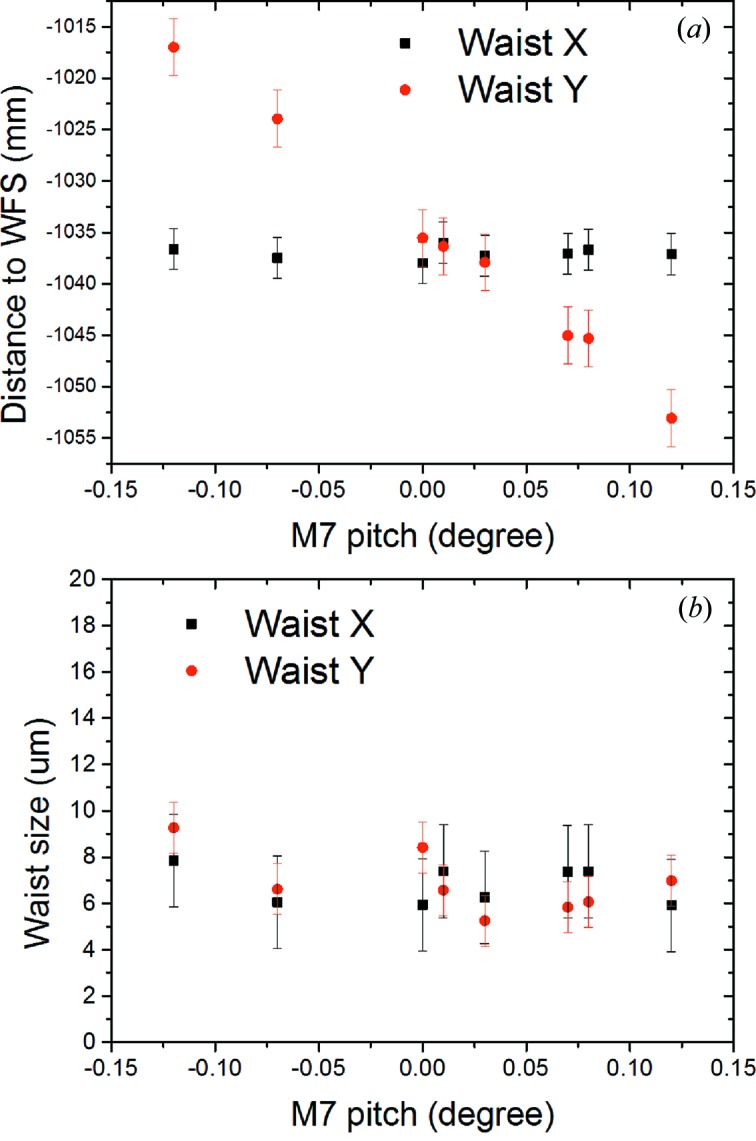
Wavefront sensor measurements of the KB focus in zero-order monochromator operation. Vertical and horizontal waist positions and sizes were measured as a function of M7 pitch angle (keeping M8 fixed). (*a*) Absolute distance of the waists to the wavefront sensor (red: vertical; black: horizontal) and (*c*) waist sizes in FWHM. Measurements were performed at 13.5 nm with the PG monochromator in zero order.

As for the ablative imprints technique, the exit slit of the PG1 monochromator was kept open during measurements in zero order. The horizontal and vertical focus sizes were reconstructed from the average of ten single-shot wavefronts recorded for each M7/M8 mirror setting. The results are shown in Fig. 11 ▸. The distance between vertical (red) and horizontal (black) waist positions and the Hartmann sensor plate are plotted as a function of M7 pitch angle (Fig. 11*a* ▸). Again, only the vertical waist position changes with M7 pitch angle. As can be seen from this figure, both horizontal and vertical focus overlap at the M7 pitch angle of 0.08°. The FWHM waist sizes are given in Fig. 11(*b*) ▸ and are (7 ± 1) µm FWHM in the vertical and (7 ± 2) µm FWHM in the horizontal direction. Note that the waist size does not change with the pitch angle. Similar results were obtained from measurements carried out by varing the pitch angle of the M8 mirror with M7 fixed. Roll and yaw angles of the mirrors as well as translation parallel to the mirror normal were also varied, which resulted in a skewed beam profile and astigmatic focusing. Considering the results from both diagnostic techniques it becomes obvious that: (i) the horizontal and vertical focus are decoupled from each other; (ii) it is possible to adjust the mirrors such that astigmatism is no longer present; (iii) the KB focus can be brought to its nominal position (zero position on the abscissa in Fig. 9 ▸). However, for geometrical reasons it was decided to set the KB focus to a position 10 mm downstream of the nominal position of 400 mm measured from the M8 mirror pole, as it provided more space to work later with the Raman spectrometer and did not affect the focal size. Astigmatism has been minimized. Furthermore, comparison of the results from the two diagnostic techniques (monochromator in zero order, exit slit open) clearly demonstrates that both approaches are consistent.

### 4.2. KB focus optimization for first-order monochromator operation   

**Figure 12:**
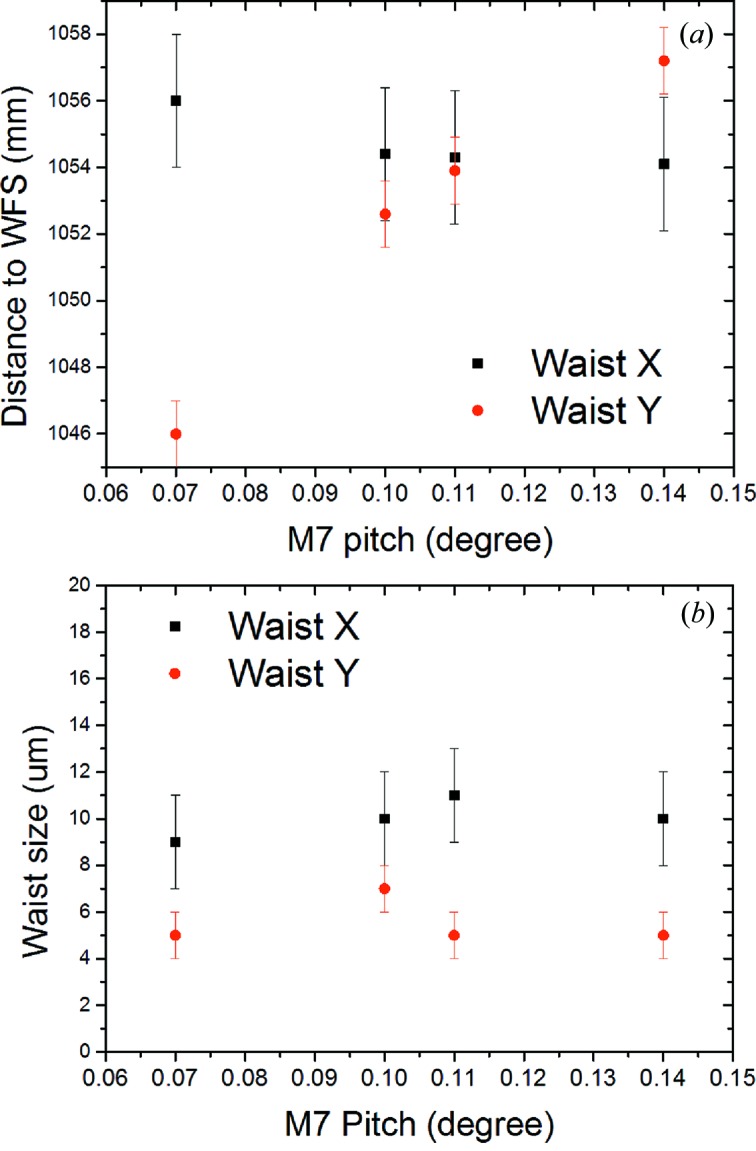
Wavefront sensor measurements of the KB focus in first-order monochromator operation. Vertical and horizontal waist positions and sizes were measured as a function of M7 pitch angle. (*a*) Absolute distance of the waists to the wavefront sensor (red: vertical; black: horizontal) and (*b*) FWHM waist sizes. Measurements were performed at 13.5 nm, the PG monochromator in first order (*c*_ff_ = 2), exit slit width 20 µm. A minimum vertical focus size of (5.8 ± 1) µm FWHM has been achieved.

**Figure 14:**
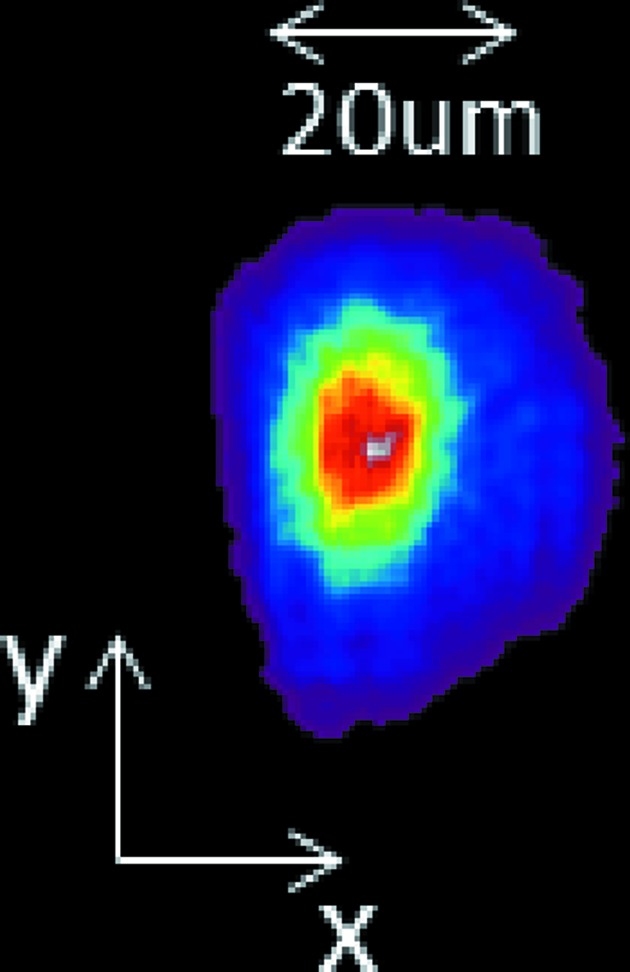
Reconstructed final spot profile from recorded wavefront.

As already mentioned, in order to achieve the ultimate Raman spectrometer resolution of up to 2 meV, the nominal operation mode of the primary monochromator for Raman scattering experiments is the dispersive high-resolution mode with narrow energy bandwidth accomplished by a small vertical exit slit width of the PG monochromator down to 20 µm. There, the exit slit basically works as a source for the KB system. This affects the KB optics focusing compared with zero-order operation and should allow to reach FEL microfocusing conditions with 4 µm FWHM (vertically). However, it makes an individual optimization using WFS necessary. The KB mirrors alignment and WFS measurements were carried out at a FEL wavelength of 13.5 nm, operating the monochromator in first diffraction order (*c*_ff_ = 2). The processed data from the WFS measurements are shown in Figs. 12 ▸ and 13 ▸ for the M7 and M8 pitch variation, respectively. A reconstruction of the final spot and its relative wavefront are shown in Figs. 14 ▸ and 15 ▸, respectively. As can be seen from the results, the KB optics provide a designed focal size of (5.8 ± 1) µm FWHM in the vertical direction and (6 ± 2) µm FWHM in the horizontal direction for the PG monochromator working in the first diffraction order. It is important to stress that the horizontal source size is not limited by the PG monochromator exit slit. Therefore it strongly depends on the FEL pointing stability and the actual source size in the undulator. The optical surface quality has a minor effect on the focus size. Ray tracing in *SHADOW* shows that including roughness and slope errors for KB focusing elements in simulations results in only a slight increase of the focal size, *i.e.* V × H = 4.1 × 6.7 µm with roughness and errors included instead of V × H = 3.8 × 5.6 µm without taking them into account. Overall the measured focii after the optimization process show excellent agreement with the simulation results.

## 5. Conclusion   

A new KB refocusing mirrors unit was developed and commissioned at the FLASH PG1 monochromator beamline. It provides microfocusing of (5.8 ± 1) µm FWHM vertically and (6 ± 2) µm FWHM in the horizontal direction at the sample position of the VUV Raman spectrometer. The focus optimization was performed by using two complementary measurement techniques, namely ablation imprints and wavefront analysis. The vertical focus achieved matches the design value of the KB pair system and by that fulfills the requirement in terms of focal spot position and waist size for the high-resolution VUV Raman spectrometer.

## Figures and Tables

**Figure 13:**
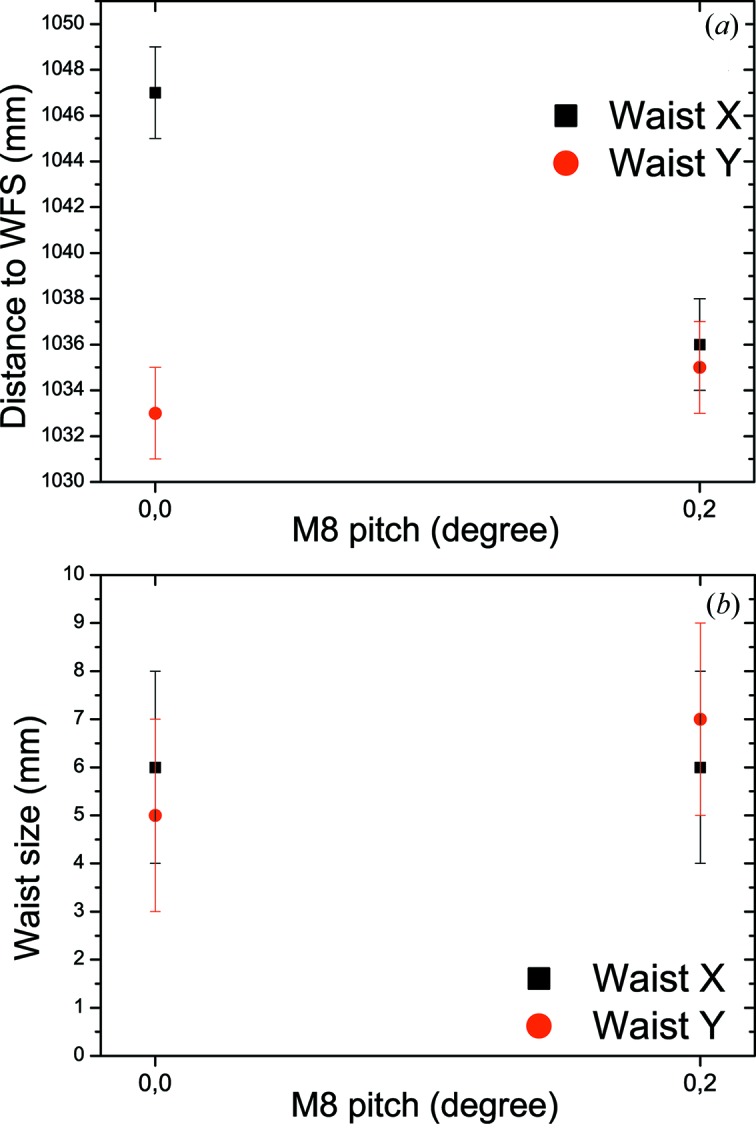
Wavefront sensor measurements of the KB focus in first-order monochromator operation. Vertical and horizontal waist size (FWHM) for two M8 pitch angles (M7 being fixed).

**Figure 15:**
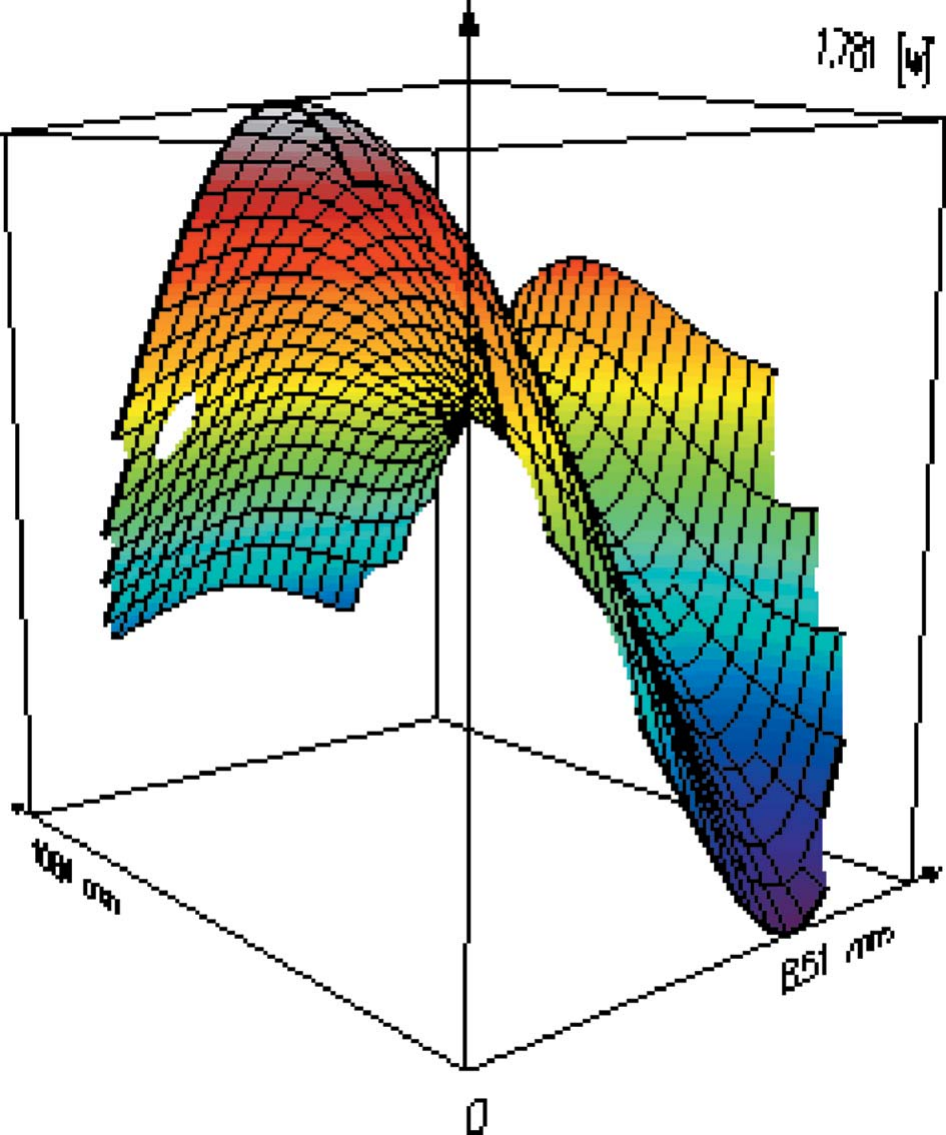
Wavefront (recorded at Hartmann plate position) for optimized KB pair optics. The peak-to-valley is given in wavelengths (1.7 nm × 14 nm).

**Table:** θ is the angle between the ellipse major semi-axis and the vector to the mirror pole in polar coordinates (the ellipse centre is the coordinate system centre). The demagnification of M7 is 4.9:1 and of M8 is 9.9:1. Tan = tangential; Sag = sagittal.

Mirror	Incidence angle (°)	Distance from source (m)	Shape	Radius (m)
M1	2	52	Torroidal	714.8/3.63 (Tan/Sag)
SMU	2	60.72	Cylindrical	0.628 (Sag)
M7	6	72.67	Elliptical	(Tan) 1.77/0.14 (θ = 5.2°)
M8	6	72.87	Elliptical	(Tan) 2.27/0.13 (θ = 2.3°)

**Table:** 

Mirror	Size (L × W) (mm)	Slope error (arcsec r.m.s.)	Microroughness (nm r.m.s.)
M1	490 × 30	0.5	0.5
SMU	280 × 30	0.5	0.37
M7	110 × 25	0.14	0.4–0.6
M8	50 × 25	0.2	0.4–0.45

**Table 2:** Results from the KB pair ray tracing The column headed ‘Influence (effect)’ shows how strong a particular DoF affects the focusing properties of the mirror and its specific effect in the ray tracing. ‘Resolution’ specifies needed resolution for the designed mirror manipulator estimated *via* *SHADOW*. ‘Travel range’ provides the specified range for the translation/angle for the mirror manipulator.

Degree of freedom	Influence (effect)	Resolution	Travel range
Translations			
Orthogonal to mirror normal	Weak (losses)	0.1 mm	±10 mm
Along beam	Moderate (astigmatism, size, losses)	0.05 mm	±10 mm
Along mirror normal	Strong (astigmatism, losses)	0.03 mm	±5 mm

Rotations			
Pitch	Strong (astigmatism, losses)	0.003°	±2°
Roll	Moderate (astigmatism, size, shape)	0.05°	±2°
Yaw	Weak (shape, size)	0.1°	±2°

## References

[bb1] Ackermann, W. *et al.* (2007). *Nat. Photon.* **1**, 336–342.

[bb2] Cerrina, F. & Sanchez del Rio, M. (2010). *Handbook of Optics*, 3rd ed., ch. 35. New York: McGraw Hill.

[bb3] Chalupský, J., Krzywinski, J., Juha, L., Hájková, V., Cihelka, J., Burian, T., Vyšín, L., Gaudin, J., Gleeson, A., Jurek, M., Khorsand, A. R., Klinger, D., Wabnitz, H., Sobierajski, R., Störmer, M., Tiedtke, K. & Toleikis, S. (2010). *Opt. Express*, **18**, 27836–27845. 10.1364/OE.18.027836 21197057

[bb4] Flöter, B., Juranić, P., Kapitzki, S., Keitel, B., Mann, K., Plönjes, E., Schäfer, B. & Tiedtke, K. (2010). *New J. Phys.* **12**, 083015.

[bb5] Gerasimova, N., Dziarzhytski, S. & Feldhaus, J. (2011). *J. Mod. Opt.* **58**, 1480–1485.

[bb6] Gerasimova, N., Dziarzhytski, S., Weigelt, H., Chalupský, J., Hájková, V., Vyšín, L. & Juha, L. (2013). *Rev. Sci. Instrum.* **84**, 065104. 10.1063/1.4807896 23822375

[bb25] Keitel, B., Plönjes, E., Kreis, S., Kuhlmann, M., Tiedtke, K., Mey, T., Schafer, B. & Mann, K. (2016). *J. Synchrotron Rad.* **23**, 43–49. 10.1107/S1600577515020354 26698044

[bb7] Kirkpatrick, P. & Baez, A. V. (1948). *J. Opt. Soc. Am.* **38**, 766–774. 10.1364/josa.38.000766 18883922

[bb8] Liu, M. (1982). *Opt. Lett.* **7**, 196–198. 10.1364/ol.7.000196 19710869

[bb9] Martins, M., Wellhöfer, M., Hoeft, J., Wurth, W., Feldhaus, J. & Follath, R. (2006). *Rev. Sci. Instrum.* **77**, 115108.

[bb10] Petersen, H. (1982). *Opt. Commun.* **40**, 402–406.

[bb11] Raimondi, L., Svetina, C., Mahne, N., Cocco, D., Capotondi, F., Pedersoli, E., Manfredda, M., Kiskinova, M., Keitel, B., Brenner, G., Plönjes, E., Mey, T., Mann, K. & Zangrando, M. (2014). *Proc. SPIE*, **9208**, 920804.

[bb12] Riemer, F. & Torge, R. (1983). *Nucl. Instrum. Methods Phys. Res.* **208**, 313–314.

[bb13] Rusydi, A., Goos, A., Binder, S., Eich, A., Botril, K., Abbamonte, P., Yu, X., Breese, M. B. H., Eisaki, H., Fujimaki, Y., Uchida, S., Guerassimova, N., Treusch, R., Feldhaus, J., Reininger, R., Klein, M. V. & Rübhausen, M. (2014). *Phys. Rev. Lett.* **113**, 067001. 10.1103/PhysRevLett.113.067001 25148343

[bb14] Schäfer, B. & Mann, K. (2002). *Appl. Opt.* **41**, 2809–2817. 10.1364/ao.41.002809 12027167

[bb15] Siewert, F., Noll, T., Schlegel, T., Zeschke, T. & Lammert, H. (2004). *AIP Conf. Proc.* **705**, 847–850.

[bb16] Siewert, F., Buchheim, J., Boutet, S., Williams, G. J., Montanez, P. A., Krzywinski, J. & Signorato, R. (2012). *Opt. Express*, **20**, 4525–4536. 10.1364/OE.20.004525 22418212

[bb17] Siewert, F., Buchheim, J., Zeschke, T., Brenner, G., Kapitzki, S. & Tiedtke, K. (2011). *Nucl. Instrum. Methods Phys. Res. A*, **635**, S52–S57.

[bb18] Siewert, F., Buchheim, J., Zeschke, T., Störmer, M., Falkenberg, G. & Sankari, R. (2014). *J. Synchrotron Rad.* **21**, 968–975. 10.1107/S1600577514016221 PMC4151678 25177985

[bb19] Siewert, F., Reininger, R., Rübhausen, M., Garrett, R., Gentle, I., Nugent, K. & Wilkins, S. (2010). *AIP Conf. Proc.* **1234**, 752–755.

[bb20] Singer, A., Sorgenfrei, F., Mancuso, A. P., Gerasimova, N., Yefanov, O. M., Gulden, J., Gorniak, T., Senkbeil, T., Sakdinawat, A., Liu, Y., Attwood, D., Dziarzhytski, S., Mai, D. D., Treusch, R., Weckert, E., Salditt, T., Rosenhahn, A., Wurth, W. & Vartanyants, I. A. (2012). *Opt. Express*, **20**, 17480–17495. 10.1364/OE.20.017480 23038301

[bb21] Tiedtke, K., Azima, A., von Bargen, N., Bittner, L., Bonfigt, S., Düsterer, S., Faatz, B., Frühling, U., Gensch, M., Gerth, C., Guerassimova, N., Hahn, U., Hans, T., Hesse, M., Honkavaar, K., Jastrow, U., Juranic, P., Kapitzki, S., Keitel, B., Kracht, T., Kuhlmann, M., Li, W. B., Martins, M., Núñez, T., Plönjes, E., Redlin, H., Saldin, E. L., Schneidmiller, E. A., Schneider, J. R., Schreiber, S., Stojanovic, N., Tavella, F., Toleikis, S., Treusch, R., Weigelt, H., Wellhöfer, M., Wabnitz, H., Yurkov, M. V. & Feldhaus, J. (2009). *New J. Phys.* **11**, 023029.

[bb22] Tono, K., Togashi, T., Inubushi, Y., Sato, T., Katayama, T., Ogawa, K., Ohashi, H., Kimura, H., Takahashi, S., Takeshita, K., Tomizawa, H., Goto, S., Ishikawa, T. & Yabashi, M. (2013). *New J. Phys.* **15**, 083035.

[bb23] Yashchuk, V. V., Morrison, G. Y., Church, M., Artemiev, N. A., Celestre, R., Domning, E. E., Howells, M., Kunz, M., McKinney, W. R., Merthe, D. J., Smith, B. V., Tamura, N. & Padmore, H. A. (2013). *J. Phys. Conf. Ser.* **425**, 152004–152008.

